# Bedside Tracheostomy for Critically Ill Pediatric Patients in the PICU: Clinical Experience in a Single Center

**DOI:** 10.3390/children12111558

**Published:** 2025-11-17

**Authors:** Young Tae Lim, Jung Eun Kwon

**Affiliations:** 1 Department of Pediatrics, School of Medicine, Kyungpook National University, Daegu 41944, Republic of Korea; ytlim@knuh.kr; 2 Division of Pediatric Cardiology, Kyungpook National University Children’s Hospital, Daegu 41404, Republic of Korea

**Keywords:** respiratory failure, child, critical care, mortality, pediatric intensive care unit, tracheostomy

## Abstract

**Background/Objectives:** Children with neurological impairments, especially those who are non-ambulatory, may require additional care services beyond what is available for the general pediatric population, and tracheostomy may be necessary for addressing respiratory problems, but no established consensus or clear guidelines have been established on the optimal timing of this procedure in the pediatric intensive care unit (PICU). **Methods:** We conducted a study involving 38 patients with neurological impairments who underwent tracheostomy in the PICU from January 2017 to December 2022. We collected demographic, tracheostomy, and outcome data and compared the data between two groups based on the duration of mechanical ventilation before tracheostomy. **Results:** The patients had heterogeneous neurological conditions, with refractory epilepsy being the most common. Almost all patients received tracheostomy for prolonged mechanical ventilation, with a median duration of 14.5 days of mechanical ventilation before the procedure. A majority of the patients (60.5%) experienced complications related to tracheostomy. The overall mortality rate was 36.8%, with 7.9% directly related to tracheostomy. When the patients were divided into two groups based on the median duration of mechanical ventilation before tracheostomy, the group that received tracheostomy earlier had significantly shorter total PICU stays and hospitalization stays compared to the group that received it later. **Conclusions:** Children with neurological impairments who undergo tracheostomy have substantial comorbidities and a high rate of complications and mortality. Earlier tracheostomy, based on shorter mechanical ventilation duration, was associated with significantly reduced PICU and hospital stay without increasing adverse outcomes. These findings suggest that timely tracheostomy may improve resource utilization in this medically fragile population.

## 1. Introduction

Children with neurological impairments may face challenges that are not present in their healthy peers. Respiratory problems are among the most common issues that children with neurological impairments may experience. Because of neurological impairments, patients who are non-ambulatory are at an increased risk for respiratory problems because of several risk factors, such as recurrent aspiration, poor airway clearance, respiratory muscle weakness, kyphoscoliosis, and sleep apnea [1]. Therefore, healthcare services that are designed for the general pediatric population may not be sufficient to address these issues, and additional care services will be necessary [2].

Tracheostomy involves creating an opening in the anterior wall of the trachea to facilitate ventilation and airway access, which is frequently performed in adult intensive care units. However, pediatric patients do not require tracheostomies as frequently as adults, with the procedure being performed in approximately 3% of pediatric and adolescent patients receiving mechanical ventilation [3]. Despite its lower frequency in the pediatric population, tracheostomy is often a necessary procedure for addressing respiratory problems in children with neurological impairments [4].

Performing tracheostomy at the bedside has been reported as a feasible and safe alternative to operating room procedures, particularly for critically ill or medically fragile patients. A recent meta-analysis comparing bedside and operating room surgeries in critically ill newborns demonstrated comparable safety and outcomes between the two settings, suggesting that bedside tracheostomy can be a reasonable option in selected pediatric patients [5].

Despite the recognized need for tracheostomy in pediatric patients with neurological impairments, no consensus or clear guidelines have been established regarding the optimal timing of this procedure for patients requiring prolonged mechanical ventilation [6]. Several factors should be considered when determining the appropriate timing of tracheostomy, including the predictability of the disease course, available expertise and resources, and parental anxiety. Therefore, this study aimed to describe the clinical characteristics and outcomes of non-ambulatory pediatric patients with neurological impairments who underwent tracheostomy in the PICU, and to assess whether the timing of tracheostomy, based on mechanical ventilation duration, affects clinical outcomes.

## 2. Materials and Methods

### 2.1. Patients and Data

We enrolled patients aged between 1 month and 18 years who were admitted to the PICU and underwent tracheostomy between January 2017 and December 2022 at a tertiary care medical institution. The inclusion criteria were limited to patients who had neurological impairments and were in a non-ambulatory state at the time of tracheostomy.

The electronic medical records were retrospectively analyzed to extract the following information: age and weight at the time of tracheostomy, sex, underlying disease, indication for tracheostomy (e.g., long term mechanical ventilation or airway obstruction), Glasgow coma scale (GCS) score on PICU admission, presence of central venous access devices and gastrostomy at the time of tracheostomy, use of inotropes at the time of tracheostomy, presence of kyphoscoliosis, failure to thrive (FTT) at the time of tracheostomy, tracheostomy-related complications, and mortality. Tracheostomy-related complications were identified through a review of postoperative clinical progress notes, nursing records, and otolaryngology consultations. Complications were classified as early (within 1–7 days) or late (>7 days). FTT was defined as a bodyweight less than the 3rd percentile for age. In this study, the growth chart published by the Korean Society of Pediatrics in 2017 was used [7]. The duration of the hospital and PICU stay and time from the procedure to discharge were calculated. Patients were categorized into early and late groups based on the median duration of mechanical ventilation before tracheostomy (14.5 days). Those ventilated for <14 days were classified as the early group, and those for ≥14 days as the late group.

### 2.2. Tracheostomy Clinical Approach

Before tracheostomy, the attending physician provided a detailed explanation to the patient’s caregivers regarding the necessity of the procedure, its safety, and potential postoperative complications. Tracheostomy was performed only after obtaining consent from the caregivers. All tracheostomies in this study were performed electively in the PICU. No patients underwent emergency tracheostomy. An experienced otolaryngologist performed a tracheostomy at the patient’s bedside in the PICU. All tracheostomies were performed as a permanent tracheostoma, with maturation sutures placed between the tracheal wall and the skin. Stay sutures (traction sutures) were not routinely used, as none of the patients were considered candidates for decannulation due to irreversible neurological impairment. The intensivist administered midazolam, ketamine, dexmedetomidine, and rocuronium as preoperative and intraoperative medications. Patients’ blood pressure, heart rate, heart rhythm, and oxygen saturation were monitored during the procedure. The patients were kept in the PICU until their vital signs were stable and their general condition was good, after which they were transferred to the general ward. All patients remained in the PICU for the first 7 postoperative days, during which daily wound dressing was performed. Careful positioning was maintained on the first postoperative day to prevent accidental decannulation, and routine otolaryngology evaluation with stitch removal was conducted on postoperative day 7. The caregivers were trained and informed about tracheostomy management by the medical staff before discharge. After discharge, tracheostomy status was regularly monitored and assessed through follow-up appointments with the pediatric and otolaryngology departments.

### 2.3. Statistical Analysis

IBM SPSS Statistics version 26 (IBM Corp., Armonk, NY, USA) was used to perform statistical analyses. Continuous variables are expressed as the medians and interquartile ranges (25th–75th percentiles), and nominal variables are expressed as numbers and percentages. Mann–Whitney U-tests were used to compare continuous variables, and the chi-square test or Fisher’s exact test was used for categorical variables. Statistical significance was set at *p* < 0.05.

### 2.4. Ethics Statement

This study was reviewed and approved by Institutional Review Board of Kyungpook National University Chilgok Hospital (IRB no. 2022-11-23). This study was conducted in accordance with the Declaration of Helsinki.

## 3. Results

### 3.1. Patient Characteristics

During the study period (January 2017 to December 2022), 56 patients aged <18 years underwent tracheostomy. Neonates admitted to the neonatal intensive care unit (n = 9) and patients without neurological impairments (n = 9) were excluded. Ultimately, the study included 38 patients who met the inclusion criteria.

Of the 38 patients included in the study, 19 (50%) were male, with a median age of 7.3 (IQR 0.9–13) years at the time of tracheostomy. The age of the patients ranged from 45 days to 17.4 years, and 10 (26.3%) patients were <1 year old. At the time of tracheostomy, the median body weight was 14.6 (IQR 8.6–19.7) kg, and 17 (44.7%) patients were in a state of FTT. Upon PICU admission, the median GCS score for the 38 patients was 10 (IQR 6.3–13.0) points. Moreover, 44.7% (17/38) of the patients had kyphoscoliosis, and 34.2% (13/38) had a gastrostomy at the time of tracheostomy. Central venous access devices were present in 73.7% (28/38) of the patients at the time of tracheostomy, and 28.9% (11/38) were receiving inotropic agents (Table 1).

**Figure 1:**
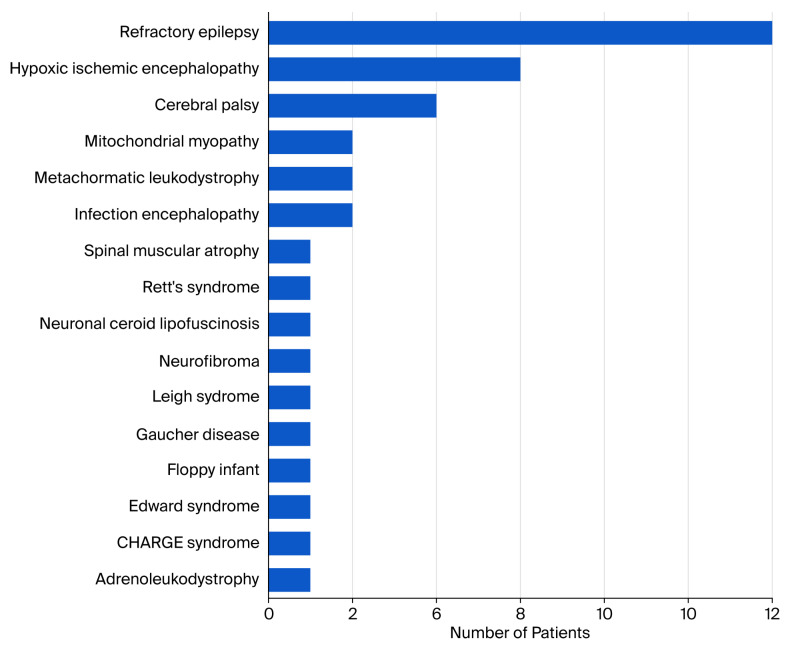
Etiology of patients’ neurological conditions.

The etiology of the patients’ neurological conditions was heterogeneous, with refractory epilepsy being the most common, in 12 (31.6%) patients, followed by hypoxic–ischemic encephalopathy (HIE) in 6 (15.8%) patients and cerebral palsy (CP) in 4 (10.5%) patients. The remaining patients were diagnosed with various conditions (Figure 1).

### 3.2. Tracheostomy Data

The majority of the patients (n = 36, 94.7%) received tracheostomy for prolonged mechanical ventilation caused by chronic respiratory failure and failed ventilator weaning. The remaining two patients received tracheostomy to manage airway obstruction. One patient had subglottic stenosis, and the other had airway obstruction due to a craniofacial anomaly. All patients had undergone intubation and mechanical ventilation before tracheostomy. The median duration of mechanical ventilation before tracheostomy was 14.5 (IQR 6.8–20) days (Table 2).

### 3.3. Outcome Data

The median length of PICU stay was 30 (IQR 19–41.5) days, and the median length of hospital stay was 42 (IQR 28.5–54) days. In addition, the median time from tracheostomy to discharge was 20.5 (IQR 15–38.8) days (Table 3).

A majority of the patients (60.5%) experienced tracheostomy-related complications. In total, 29 complications were identified in 23 patients (Table 3), with 4 occurring within 7 days of tracheostomy (early complications) and the remaining 25 occurring after 7 days of tracheostomy (late complications). The most frequent complication was granulation tissue formation, which was observed in 15 patients (52%). None of these cases required surgical intervention, and all were managed with topical treatment. Other complications included accidental decannulation (4), wound ulceration (3), bleeding (3), pressure sores (2), and tube occlusion (1). Three cases resulted in serious consequences or death. One early complication was accidental decannulation, which occurred 48 h after tracheostomy, resulting in the patient’s death as the tube was not properly reinserted. The remaining two cases were delayed complications: one patient required surgery for the brachiocephalic artery tear during tube replacement and the other patient died from accidental decannulation while at home.

In this study, the overall mortality rate was 36.8% (14 of 38 patients). Of these, three deaths (7.9%) were directly related to tracheostomy, whereas the remaining deaths were attributed to the underlying medical conditions of the patients (Table 3). Among the tracheostomy-related deaths, the first case was caused by accidental decannulation that occurred 48 h after the procedure. The second case was caused by massive bleeding that occurred during a routine outpatient visit, which was confirmed by neck computed tomography to be caused by an injury to the brachiocephalic artery. A vascular surgeon inserted the stent at the lesion site. Approximately 3 months later, the patient experienced another episode of massive bleeding through the tracheostomy and died from emergency treatment not being available, with the previous injury to the artery suspected as the cause. The last patient died from accidental decannulation that occurred while the patient was at home. Four of the deceased patients died while undergoing observation and treatment after tracheostomy, while the remaining ten died during follow-up after being discharged from the hospital after tracheostomy.

**Figure 2:**
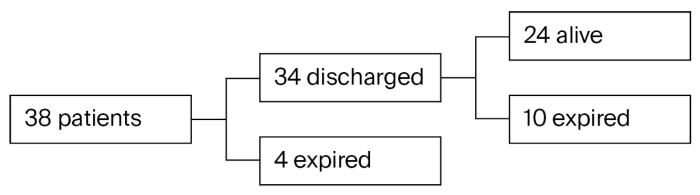
Outcomes.

Among the 38 patients who underwent tracheostomy, 4 expired while undergoing observation and treatment at the hospital, whereas 34 were discharged in a satisfactory general condition. Following hospital discharge, 10 of these patients died during follow-up through regular outpatient treatment, whereas the remaining 24 are currently alive (Figure 2). None of the patients underwent decannulation, and all surviving patients continued to perform home ventilation through tracheostomy.

### 3.4. Comparison of Groups by Duration of Mechanical Ventilation Before Tracheostomy

In this study, the median duration of mechanical ventilation before tracheostomy was 14.5 (IQR 6.8–20) days. To further investigate the effect of mechanical ventilation duration on patient outcomes, the patients were divided into two groups based on the median duration. Group A consisted of patients who received mechanical ventilation for <14 days, whereas group B consisted of patients who received mechanical ventilation for >14 days. Statistically significant differences in the total PICU stay and hospitalization stay were found between the two groups, with group A having shorter times for both factors (*p* < 0.001 and 0.0003, respectively). The study also examined other patient characteristics and found no differences between the two groups in terms of sex, tracheostomy indications, age at tracheostomy, weight at tracheostomy, GCS score upon PICU admission, presence of central venous access devices at the time of tracheostomy, use of inotropes at the time of tracheostomy, presence of kyphoscoliosis and FTT at the time of tracheostomy, time from the procedure to discharge, complications, or mortality (Table 4). Among the three tracheostomy-related mortalities, two occurred in the early tracheostomy group and one in the late group; however, given the very small number of severe events, this difference is unlikely to be clinically meaningful. Overall, there were no clear differences in the types or severity of tracheostomy-related complications between the early and late groups.

## 4. Discussion

Compared with past procedures, significant changes have been made in the current indications for pediatric tracheostomy. Previously, upper airway obstructions caused by viral and bacterial infections such as croup, diphtheria, and epiglottitis were the most common indications for pediatric tracheostomy. However, with the development of vaccines and antibiotics, the incidence of these infections causing airway obstruction has decreased [8]. In addition, advances in critical care have improved the survival rate of pediatric patients with critical illness requiring prolonged respiratory support, including premature infants [3]. As a result, the indications for pediatric tracheostomy have shifted. A Canadian study of pediatric tracheostomy over 30 years found that the number of infection-related tracheostomies decreased, whereas those due to neurological impairments increased significantly [9]. Similarly, a 20-year study of tracheostomies in the PICU in India found that upper airway obstruction was the most common indication in the first 10 years, whereas central neurological impairment was the most common indication in the latter 10 years [10]. Currently, the most frequent indications for tracheostomy in children are congenital upper airway anomalies causing airway obstruction or prolonged mechanical ventilation due to respiratory failure.

Neurological diseases leading to a non-ambulatory state often require prolonged mechanical ventilation. Children with neurological impairments are more susceptible to respiratory insufficiency than their healthy counterparts. Malnutrition-related respiratory muscle wasting in the presence of neurological impairments leads to ineffective breathing, and frequent aspiration and decreased cough efficacy with retention of secretions increases the likelihood of recurrent and chronic lung infections. Hypoxemia due to obstructive sleep apnea syndrome is also common in these patients. Thus, children with neurological impairments are at risk of respiratory failure and often require respiratory support such as mechanical ventilation [1,11]. Tracheostomy may offer specific advantages to children with neurological impairments who are dependent on ventilation. It can reduce the sedation requirement, increase the duration of awake and interactive periods with caregivers, and facilitate discharge from the PICU to an appropriate supported environment, such as home, rather than hospital [12]. Therefore, tracheostomy should be considered in children with neurological impairments requiring prolonged mechanical ventilation.

This study investigated the characteristics of pediatric patients with neurological impairments who underwent tracheostomy. The patients were typically non-ambulatory because of underlying diseases, with refractory epilepsy being the most common, followed by HIE and CP (Figure 1). Upon hospital admission, the median GCS score was 10 (IQR 6.3–13) points, indicating moderate brain injury. Nearly half of the patients had kyphoscoliosis and FTT at the time of tracheostomy, and some patients also had gastrostomy. In addition, the majority of patients who received tracheostomy had central venous access, and nearly all patients underwent tracheostomy for prolonged mechanical ventilation. Given the scarcity of studies specifically investigating pediatric tracheostomy in patients with neurological impairments, the information presented here can help identify the characteristics of this patient population.

No consensus or guidelines have been established for the appropriate timing for tracheostomy in children with neurological impairments who require prolonged mechanical ventilation in the PICU. Wakeham et al. [13] conducted a study on tracheostomy use in 82 North American PICUs and found significant variations in both the rate and timing of tracheostomy use across different units. This variability appears to be influenced by local PICU characteristics, available resources, unit practice patterns, and practitioner attitudes toward tracheostomy. Establishing a consensus and guideline for pediatric tracheostomy timing is challenging because of the heterogeneity and complexity of potential risks associated with underlying diseases in patients requiring tracheostomy. Several recent studies have investigated the optimal timing for tracheostomy in pediatric patients requiring prolonged mechanical ventilation in the PICU [6,14,15]. All these studies compared and analyzed early and late groups based on an intubation period of 14 days, and similar results were obtained. Early tracheostomy was found to be associated with lower rates of complications, higher rates of successful weaning, and decreased utilization of intensive care resources. Moreover, it was linked to a reduction in the duration of mechanical ventilation, length of PICU stay, and overall hospital stay. The underlying diseases of the patients included in the above three studies were heterogeneous. In this study of patients with neurological conditions, the median duration of mechanical ventilation before tracheostomy was 14.5 (IQR, 6.8–20) days. To compare the outcomes of early versus late tracheostomy, patients were divided into two groups based on the median value, and a comparative analysis was conducted. A statistically significant difference was found between the early and late groups in terms of total hospital stay and length of PICU stay, with both being shorter in the early group (*p* = 0.003, *p* < 0.001). However, no significant difference was found in the complication or mortality rate between the two groups. These findings provide valuable insights into the effect of mechanical ventilation duration on patient outcomes and suggest that shorter durations of mechanical ventilation may be associated with shorter total PICU and hospital stay.

In this study, which focused solely on patients with neurologic diseases, tracheostomy-related complications occurred in approximately two-thirds of the patients (23/38, 60.5%). Granuloma formation was the most commonly observed complication, and none of the cases required surgical removal. Most of these complications were detected during the regular follow-up and were treated topically. A recently published systematic review of tracheostomy-related complications in children found an average complication rate of 40%, with rates varying based on factors such as age, birthweight, prematurity, comorbidities, and whether the procedure was elective or performed as an emergency step. Consistent with our study, the most common complications reported were granulomas and cutaneous lesions [16]. Another cross-sectional analysis of pediatric tracheostomy-related complications examined a total of 5309 tracheostomies and reported a complication rate of 8% [17]. Recent single-center studies have reported complication rates ranging from 25% to 55% [18,19,20,21], which may vary depending on the characteristics of the underlying disease or patient age. The study participants were patients who required tracheostomy for home ventilation and did not require decannulation, indicating that tracheostomy was necessary in the long term. Given this, the complication rate observed in our study is expected to be higher than that in other studies. Therefore, active collaboration between healthcare providers and caregivers is essential to prevent tracheostomy-related complications in patients with neurological impairments. When compared with multicenter cohort data, the complication rate in our study (60.5%) lies at the higher end of the reported pediatric tracheostomy range (25–55%). This may reflect the high medical complexity and long-term ventilator dependence of non-ambulatory neurologically impaired children. In contrast, our tracheostomy-related mortality rate (7.9%) and overall mortality rate (36.8%) fall within the ranges observed in large multicenter studies. These comparisons suggest that our patient population is more medically fragile than general pediatric cohorts, yet the outcomes remain broadly consistent with previously reported data.

It is also important to recognize that children with severe, non-ambulatory neurological impairments rarely achieve functional recovery and therefore have minimal potential for decannulation. In this population, tracheostomy is not a temporary bridging intervention but rather a long-term airway strategy that requires ongoing multidisciplinary care and vigilant prevention of complications. As a result, clinical decision-making should prioritize patient stability, reduction in PICU and hospital resource utilization, and timely transition to a supported environment, rather than expectations for eventual decannulation. Clear communication of these long-term expectations with the caregiver is essential for shared decision-making and planning for chronic care needs. Although caregiver expectations may sometimes influence surgeons to consider a temporary tracheostomy in hopes of future decannulation, such an approach is unlikely to benefit children with irreversible neurological impairment. In this population, the likelihood of successful decannulation is extremely low, and accidental decannulation can result in life-threatening airway compromise. Accordingly, the formation of a permanent tracheostoma provides a more stable and secure airway tract and is generally a safer and more appropriate approach for medically fragile, non-ambulatory patients.

The mortality rate can vary depending on the composition of the study participants. Recent single-center studies with heterogeneous patient etiologies reported an overall mortality rate of 17–37% and a tracheostomy-related mortality rate of 1.2–27% [19,20,22,23]. In this study, the overall mortality and tracheostomy-related mortality rates were 36.8% and 7.9%, respectively, which is consistent with the findings of other single-center studies. In a study involving 917 children who underwent tracheostomy, the relationship between comorbid clinical conditions and mortality was analyzed. The study found that mortality rates and hospital-resource utilization were higher in cases with neurological impairments [5]. All tracheostomies in this study were performed at the bedside. Most of the observed mortality appears to be attributable to the severity of patients’ underlying neurological conditions, rather than the procedure itself. This finding is consistent with a recent meta-analysis, which reported higher mortality in patients who underwent procedures at the bedside compared to those in the operating room, but attributed this difference primarily to the severity of the patients’ underlying medical conditions rather than the procedural setting [24]. Furthermore, other studies have reported that severe neurocognitive disability and seizures were associated with high mortality rates [21,22]. Two tracheostomy-related deaths were attributed to accidental decannulation, which could have been prevented with timely and appropriate intervention by medical staff. Patients with neurologic diseases are at a higher risk of mortality following tracheostomy than other disease groups, underscoring the need for vigilant attention and care from both medical staff and caregivers.

This study has several limitations that should be considered when interpreting the findings. First, it was a retrospective analysis of medical records from a single institution, which raises the possibility of missing data. Second, the sample size was relatively small, which limited the statistical power of the Cox proportional hazards regression analysis used to identify mortality-related risk factors. Therefore, more robust and reliable results can be obtained through a prospective multicenter study that overcomes these limitations. However, unlike other single-center studies that focus on a heterogeneous population, this study exclusively includes non-ambulatory patients with neurological impairments, providing unique insights into their tracheostomy use and characteristics. In particular, decisions regarding the timing of tracheostomy and approaches to perioperative management may reflect institution-specific practices, potentially limiting the generalizability of our findings. Future multicenter prospective studies are needed to confirm these findings and further clarify the optimal timing of tracheostomy in this medically complex population.

## 5. Conclusions

This study showed that non-ambulatory children with neurological impairments undergoing tracheostomy in the PICU have substantial comorbidities and a high rate of tracheostomy-related complications. Early tracheostomy was associated with shorter PICU and hospital stays, without differences in complications or mortality. These findings suggest that earlier tracheostomy may reduce resource utilization in this medically fragile population. Ongoing multidisciplinary care remains essential due to the high prevalence of comorbidities and tracheostomy-related complications.

## Figures and Tables

**Table 1:** Demographics of the patients (n = 38).

Variable	Value
Male	19 (50)
Age at tracheostomy (years)	7.3 (0.9–13)
Weight at tracheostomy (kg)	14.6 (8.6–19.7)
GCS score at PICU admission	10 (6.3–13)
Kyphoscoliosis	17 (44.7)
FTT	17 (44.7)
Gastrostomy	13 (34.2)
Central venous access	28 (73.7)
Inotrope use	11 (28.9)

Values are presented as numbers (%) or medians (interquartile ranges). FTT, failure to thrive; GCS, Glasgow Coma Scale; PICU, pediatric intensive care unit.

**Table 2:** Data relating to tracheostomy (n = 38).

Variable	Value
Tracheostomy indications	
Prolonged mechanical ventilation	36 (94.7)
Airway obstruction	2 (5.3)
MV before tracheostomy	38 (100)
MV duration before tracheostomy (day)	14.5 (6.8–20)
Decannulation of tracheostomy	0 (0)

Values are presented as a number (%) or median (interquartile range). MV, mechanical ventilation.

**Table 3:** Data-related outcomes (n = 38).

Variable	Value
Length of PICU stay (day)	30 (19–41.5)
Time from tracheostomy to discharge (day)	20.5 (15–38.8)
Total hospital stay (day)	42 (28.5–54)
Number of tracheostomy-related complications	29
Early complications	4 (13.8)
Late complications	25 (86.2)
Mortality after tracheostomy	14 (36.8)
Tracheostomy-related mortality	3 (7.9)

Values are presented as a number (%) or median (interquartile range). PICU, pediatric intensive care unit.

**Table 4:** Comparison of groups divided by the mechanical ventilation application period before tracheostomy.

Variable	Group A (n = 19)	Group B (n = 19)	*p*-Value
Sex			
Male	7 (36.8)	12 (63.2)	0.105
Female	12 (63.2)	7 (36.8)	
Indications for tracheostomy			
Prolonged mechanical ventilation	17 (89.5)	19 (100)	0.486
Airway obstruction	2 (10.5)	0 (0)	
Age at tracheostomy (years)	8.1 (1.5–12.9)	4.7 (1.1–12.4)	0.43
Weight at tracheostomy (kg)	14.2 (9.7–17.1)	17 (8.7–22.9)	0.54
Gastrostomy	10 (52.6)	3 (15.8)	0.017
Central venous line	13 (68.4)	15 (78.9)	0.461
Inotrope use	5 (26.3)	6 (31.6)	0.721
GCS score	11 (7.5–15)	9 (6–11.5)	0.076
Kyphoscoliosis	11 (57.9)	6 (31.6)	0.103
FTT	6 (31.6)	11 (57.9)	0.103
Complications	13 (68.4)	10 (52.6)	0.319
Death	8 (42.1)	6 (31.6)	0.501
Total hospital stay (day)	30 (22–44)	51 (41.5–76)	0.003
Total PICU stay (day)	19 (17.5–25.5)	40 (31.5–42.5)	<0.001
Time from the procedure to discharge (day)	19 (14–33.5)	24 (17.5–44.5)	0.184

Values are presented as numbers (%) or medians (interquartile ranges). GCS, Glasgow Coma Scale; FTT, failure to thrive; PICU, pediatric intensive care unit.

## Data Availability

This study is a retrospective analysis based on medical records from pediatric patients. The data presented in this study are available on request from the corresponding author due to ethical and privacy restrictions.

## Abbreviations

PICU	Pediatric Intensive Care Unit
GCS	Glasgow Coma Scale
FTT	Failure To Thrive
HIE	Hypoxic–Ischemic Encephalopathy
